# Multi-omics characterization of the necrotrophic mycoparasite *Saccharomycopsis schoenii*

**DOI:** 10.1371/journal.ppat.1007692

**Published:** 2019-05-09

**Authors:** Klara Junker, Anna Chailyan, Ana Hesselbart, Jochen Forster, Jürgen Wendland

**Affiliations:** 1 Yeast & Fermentation, Carlsberg Research Laboratory, Copenhagen, Denmark; 2 Functional Yeast Genomics, Vrije Universiteit Brussel, Brussels, Belgium; University of Melbourne, AUSTRALIA

## Abstract

Pathogenic yeasts and fungi are an increasing global healthcare burden, but discovery of novel antifungal agents is slow. The mycoparasitic yeast *Saccharomycopsis schoenii* was recently demonstrated to be able to kill the emerging multi-drug resistant yeast pathogen *Candida auris*. However, the molecular mechanisms involved in the predatory activity of *S*. *schoenii* have not been explored. To this end, we *de novo* sequenced, assembled and annotated a draft genome of *S*. *schoenii*. Using proteomics, we confirmed that *Saccharomycopsis* yeasts have reassigned the CTG codon and translate CTG into serine instead of leucine. Further, we confirmed an absence of all genes from the sulfate assimilation pathway in the genome of *S*. *schoenii*, and detected the expansion of several gene families, including aspartic proteases. Using *Saccharomyces cerevisiae* as a model prey cell, we honed in on the timing and nutritional conditions under which *S*. *schoenii* kills prey cells. We found that a general nutrition limitation, not a specific methionine deficiency, triggered predatory activity. Nevertheless, by means of genome-wide transcriptome analysis we observed dramatic responses to methionine deprivation, which were alleviated when *S*. *cerevisiae* was available as prey, and therefore postulate that *S*. *schoenii* acquired methionine from its prey cells. During predation, both proteomic and transcriptomic analyses revealed that *S*. *schoenii* highly upregulated and translated aspartic protease genes, probably used to break down prey cell walls. With these fundamental insights into the predatory behavior of *S*. *schoenii*, we open up for further exploitation of this yeast as a biocontrol yeast and/or source for novel antifungal agents.

## Author summary

Fungal infections both in humans and in crop plants are on the rise, but novel strategies to combat yeast pathogens are few and far between. The mycoparasitic yeast *Saccharomycopsis schoenii* has recently been described as being able to physically attack and kill various clinically relevant *Candida* species, including multi-drug resistant *Candida auris*. However, the molecular mechanisms of this unique predatory behavior have not been described. Here, we visualize the yeast attack using time lapse microscopy and explore nutritional conditions that trigger predatory behavior. We also use omics to discover genes and proteins upregulated by *S*. *schoenii* as it predates on the model prey *Saccharomyces cerevisiae*. We found that predation was induced by nutrient limitation and not, as we had hypothesized, linked to a special organic sulfur requirement in *S*. *schoenii*. However, an absence of organic sulfur sets off drastic transcriptional responses linked to methionine salvaging and scavenging, that are mitigated if predation is enabled during the same conditions. We found that a gene family of aspartic proteases has been expanded in *S*. *schoenii*, and that several of these genes are overexpressed during predation. In summary, our study provides new avenues to explore in the battle against fungal pathogens.

## Introduction

The burden of fungal pathogens of plants, animals and humans is increasing at an unprecedented rate [1]. Fungal diseases currently affect more than one billion people and cause over 1.6 million deaths per year [2]. The increase of immunocompromised patients in hospital has created a breeding ground for multi-drug resistant fungal infections, most severely exemplified by the yeast *Candida auris* [3]. In parallel, the usefulness of fungi range from being sources of fundamental antimicrobial agents to being used as live agricultural biocontrol agents [4,5]. Recent advances in next generation sequencing (NGS) and multi-omic methods have revolutionized the overall understanding of both pathogenic and beneficial fungi, by enabling more in-depth studies of fungal parasitic systems and opening up the exploration of novel antifungal mechanisms from lesser-known fungi [6–10]. For instance, several biotrophic fungi, i.e. fungi able to derive nutrients from their host without killing them, have lost genes in pathways for synthesis of molecules that they can readily acquire from their host.

Mycoparasitic fungi are fungi that parasitize on other fungal species, and several mycoparasitic filamentous fungi are well studied because of their abilities to physically attack plant pathogenic fungi [11–13]. Mycoparasitic fungi that kill their fungal host are called necrotrophic, and predatory organisms are organisms that consume the organism they kill [14]. Yeasts in the *Saccharomycopsis* clade, are unique necrotrophic mycoparasites in that they are single celled yeasts with the ability to physically attack and kill, and presumably predate on, a wide range of yeasts [15]. *Saccharomycopsis* yeasts are not well studied, but their mode of action involves invading fungal prey cells with small haustoria-like penetration pegs, which ultimately kill the prey cells [16]. One of the most ferocious predators in this clade, *S*. *schoenii*, has the potential as a medical biocontrol yeast as it was recently demonstrated to attack and kill several clinical isolates of pathogenic *Candida* species *in vitro*, including multi-drug resistant isolates of *C*. *auris* [17].

*Saccharomycopsis* yeasts are closely related to *Ascoidea rubescens* [18] and *Wickerhamomyces anomalus* [19] and were recently proposed to belong to a subclade of the CTG clade yeasts [20]. Virtually all organisms translate the CTG codon to leucine, typically using a tRNA^Leu^(CAG), but yeasts in the CTG clade, such as *Candida albicans*, use a modified tRNA^Ser^(CAG) to translate CTG to serine [21, 22]. Three *Saccharomycopsis* yeasts were found to harbor both a tRNA^Ser^(CAG) and a tRNA^Leu^(CAG) in their genome, still, only *Saccharomycopsis capsularis* translated the CTG codon to serine.

Organic sulfur is an essential element for all types of cells, and one of the two sulfur containing amino acids, methionine, is also one of the most energetically expensive amino acid for yeasts to produce [23]. The vast majority of microorganisms, including yeast, are able to take up sulfur in the form of inorganic sulfate and reduce it in the sulfate assimilation pathway [24], but yeasts in the *Saccharomycopsis* clade share a rare inability to assimilate inorganic sulfur [25, 26]. We recently reported that in *Saccharomycopsis fodiens* [27] and *Saccharomycopsis fermentans* [28], the genomic basis for this rare organic sulfur auxotrophy is a complete absence of all genes in the sulfate assimilation pathway.

Specific auxotrophies are often involved in parasitic behavior in both yeast and fungi [29, 30]. The human pathogenic yeast *Candida glabrata* is a nicotinic acid auxotroph, and when nicotinic acid is absent in the urinary tract of its host, such as when a catheter is used, *C*. *glabrata* becomes virulent and colonizes host tissue [31]. Similarly, the rust fungi *Puccinia graminis* have deficiencies in both the nitrate and sulfate assimilation pathways, which might have enabled or adapted it to life as an obligate biotroph [32]. In preliminary studies, Lachance *et al*. demonstrated that some *Saccharomycopsis* species appeared to change their predatory behavior depending on methionine availability, but the results were inconclusive [25].

In fungal parasites, gene loss often come hand in hand with gene and genome expansion [33, 34]. Transposable elements (TE) for instance, that shape eukaryotic genomes, are often expanded in biotrophic fungi [29, 35, 36]. TEs such as retrotransposons have ancient RNAi-mediated mechanisms, can be involved in genome defense and some appear activated during stressful conditions [37]. Gene expansion is also often coupled to genes that enable parasitic behavior [34]. For instance, the genomes of *Trichoderma* species harbor expanded gene families also including genes coding for cell wall degrading enzymes, such as chitinases, glucanases and proteases [10, 38, 39]. Several Trichoderma species are used as biocontrol agents, and upregulate and release proteases prior to their antagonism of plant-pathogenic fungi, whereas chitinases and glucanases are upregulated during active mycoparasitism [5].

*Saccharomycopsis* species have been successfully trialed as agricultural or food biocontrol agents; *Saccharomycopsis schoenii* against plant pathogens on oranges [40], and *Saccharomycopsis fibuligera* against toxic molds on speck [41]. The genome of *S*. *fibuligera* was recently sequenced, and its transcriptomic responses to organic sulfur starvation was studied [42]. To our knowledge, only one successful genetic manipulation of a *Saccharomycopsis* yeast has been achieved, but the disruption of a single protease gene in a mutagenized *S*. *fibuligera* strain was not tested in the context of predatory behavior [43].

Here we hypothesized that by taking a genomic, transcriptomic and proteomic approach, and coupling it with quantifiable predatory behavior, we could identify and separate major genetic pathways involved in starvation and predation responses in *S*. *schoenii*, with little bias. We further hypothesized that the need for organic sulfur compounds, especially methionine, plays a central role in the predatory behavior of *S*. *schoenii*. Our aim was to expose the genetic toolkit of *S*. *schoenii*, particularly in regards to its predatory behavior, with the purpose of gaining fundamental insights into its potential usefulness as a biocontrol yeast.

## Materials and methods

### Strains and culture conditions

Wild-type *Saccharomycopsis schoenii* (CBS 7425, CBS-KNAW collection, Utrecht, Netherlands) was provided by Marc-André Lachance. *Saccharomyces cerevisiae* strain BY4741 (EUROSCARF), *S*. *cerevisiae* with hygromycin resistance (BY4741; *MAT**a**; his3Δ1; leu2Δ0; met1Δ0; ura3Δ0; HSP104-GFP:hygMX* [44] Carlsberg Research Laboratory, Denmark) and *S*. *cerevisiae* H4-GFP (BY4741, *HHF1-GFP*, Carlsberg Research Laboratory, Denmark) were used as model prey cells as indicated. Yeast cells were cultured to log phase in standard medium (YPD; 10 g/L yeast extract, 20 g/L Bacto peptone, 20 g/L glucose), at 30°C, rotating. *S*. *schoenii* and *S*. *cerevisiae* were subsequently cultured alone or co-cultured as indicated on solid YPD, Nutrient limited media (CSM; 0.79 g/L Complete Supplement Mixture, 6.7 g/L Yeast Nitrogen Base (YNB) w/o amino acids with ammonium sulfate, 20 g/L glucose), Methionine deprived media (CSM-Met; 0.75 g/L Complete Supplement Mixture -Methionine, 6.7 g/L YNB w/o amino acids with ammonium sulfate 20 g/L glucose) or Starvation media (SD; 20g/L glucose 6.7 g/L YNB w/o amino acids with ammonium sulfate). Media were solidified with 10 g/L agarose for microscopy and with 20 g/L bacto-agar for cultures for genome preparation, protein preparation and RNA preparation.

### Microscopy

Differential interference contrast (DIC) and fluorescence microscopy were performed with a Zeiss Axio Imager M2 Microscope, using a halogen lamp for transmitted-light and UV for fluorescence imaging, and the software Metamorph for image acquisition. Predation quantification analyses, performed in duplicates, were performed by initially seeding co-cultured cells on several agarose pads with appropriate media, imaging new slides at each hour for six hours. Three frames, representative of the whole slide were captured. Locations where cells were growing on top of each other were selected against, in favor of locations where individual cells could be distinguished. *S*. *cerevisiae* prey cells were scored as live/non-predated (regular, round morphology), dead/non-predated (flattened, shrunken, no physical contact with *S*. *schoenii* cells) or dead/predated (vacuolarized or flattened, shrunken and in physical contact with *S*. *schoenii* cells). *S*. *schoenii* cells were scored as live (regular, full morphology) or dead (shrunken and/or flattened). The software FIJI/ImageJ was used for image processing and analysis [45]. For movies, drift in frames was corrected with the macro NMS fixTranslation v1 [46] and the plugin Image Stabiliser [47]. Cells were counted with the plugin Cell Counter and area measurements of *S*. *cerevisiae* were performed by tracing cell outlines using the elliptical selection tool.

### Genome sequencing

For genomic sequencing with Illumina MiSeq, DNA extraction and sequencing were performed by LGC Genomics (Berlin, Germany), generating a 250-bp paired-end library and a 8-kb mate-pair library. For genomic PacBio sequencing, DNA was prepared using the QIAGEN Blood & Cell Culture DNA Maxi Kit with a QIAGEN Genomic-tip 500/G (QIAGEN GmbH, Hilden, Germany) according to the manufacturer’s protocol. Subsequent PacBio sequencing, based on Single Molecule Real-Time (SMRT) technology, was performed by DNA Link Inc. (Seoul, Republic of Korea), using kits and reagents from Pacific Biociences. Quality controlled genomic DNA was used to prepare the SMRTbell library and fragments smaller than 20kb were removed using the Blue Pippin Size selection system. Polymerase-SMRTbell-adaptor complexes were loaded into four SMRT cells and sequenced using C4 chemistry (DNA sequencing Reagent 4.0). 240-minute movies were captured for each SMRT cell using the PacBio RS II sequencing platform, generating one set of raw sequencing subreads per SMRT cell.

### Genome assembly

Raw PacBio sequencing subreads were filtered on quality and length, using the RS_subreads protocol in PacBio’s SMRT-Portal software, run through Amazon Web Services Inc (Seattle, USA) and exported as fastq files. Filtered subreads were batch error-corrected using the tool “Correct PacBio Reads (beta)” and then *de novo* assembled using the tool “*De Novo* Assemble PacBio Reads (beta)” in CLC genomics workbench v.9.5 (QIAGEN Aarhus, Aarhus C, Denmark), generating 78 contigs. At this stage the assembly was quality controlled with QUAST analysis [48]. The 78 contigs were then further polished using Illumina MiSeq generated 250-bp paired-end library and 8-kb mate-pair library, resulting in a final draft genome assembly of 29 contigs with 14.3 mega base pairs. Our draft genome assembly is available in S1 Material.

### Genome annotation

Open Reading Frames (ORFs) were predicted as sequences with an AUG start codon, spanning >300 bp. ORFs translated with the Alternative Yeast Nuclear Code (AYNC) were subjected to a cloud-based blastx against the non-redundant protein database (nr), deposited by 06.06.2017, using the plugin Blast2GO [49] inside the software CLC genomics v9.5. To functionally annotate the *S*. *schoenii* ORFs, all ORFs were subjected to blastx strategies against proteins from *S*. *cerevisiae*, *C*. *albicans* or both, and homology was inferred as a hit with a bit score >55 [50]. Overlapping ORFs were removed manually, resulting in 4,660 annotated genes. Our genome annotation of *S*. *schoenii* is available in S2 Material and S1 Data. tRNA genes were identified using tRNAScan-SE [51].

### Proteomic analysis

For the proteomic analysis, *S*. *schoenii* cells were cultured for three hours under three different conditions; on YPD media alone (“standard”), on SD media alone (“starvation”) or on SD media together with equal numbers of *S*. *cerevisiae* (“predation”). Cells were pelleted and flash frozen in liquid nitrogen. Subsequent proteomic analysis was performed by Phylogene (Bernis, France). Proteins were extracted, purified and concentration of lysates was determined by Pierce 660 nm assay. Peptides were prepared according to the FASP method, ultrafiltered, reduced, alkylated and digested by trypsin. Peptides were purified by SPE chromatography and peptide concentration was determined using the BCA method. Liquid chromatography-tandem mass spectrometry (LC-MS/MS) measurements were done in triplicates. Chromatography was performed using Ultimate 3000 (Dionex) and data acquired using the Q-Exactive Plus (Thermo) mass spectrometer. Proteins were identified using SEQUEST-HT algorithm against two custom databases with *S*. *schoenii* ORFs translated with Standard Code or Alternative Yeast Translation Code respectively, and when *S*. *schoenii* and *S*. *cerevisiae* were co-cultured, also against a database containing reference proteome of *S*. *cerevisiae* mined from UNIPROT. Data were processed using Minora and feature mapper for Proteome Discoverer 2.2 software. Statistical analyses were performed by using Precursors Ions quantifier node for Proteome Discoverer 2.2 software. Abundances of *S*. *schoenii* peptides and proteins were measured against the abundances of *S*. *schoenii* peptides and proteins when cultured alone on YPD. Only hits identified to non-overlapping ORFs with a homolog in other yeasts were analyzed further. The mass spectrometry proteomics data have been deposited to the ProteomeXchange Consortium [52] via the PRIDE partner repository [53] with the dataset identifier PXD008453, with a summary in S1 Data.

### Transcriptomic analysis

For the transcriptomic analysis, RNA was extracted from *S*. *schoenii* cells were cultured alone or co-cultured with equal amounts of *S*. *cerevisiae* cells for three hours on YPD, CSM, CSM-Met or SD media. Each condition was performed in three biological replicates. RNA was extracted using the RiboPure-Yeast Kit (Ambion, Thermo Fisher Scientific) and DNAse treated. BGI Europe A/S, (Copenhagen, Denmark) performed quality control, constructed polyA-selected strand-specific transcriptomic libraries, and sequenced the samples at 10 Mb clean reads/sample using Illumina Hiseq4000 PE100. Raw reads from each triplicate were pooled, mapped to ORFs in the *S*. *schoenii* genome and normalized by total reads per sample using CLC genomics workbench v.9.5. Transcription was expressed as mean normalized expression values. To exclude noise, only genes with transcription values of >100 and a 10-fold upregulation during any of the conditions, compared to *S*. *schoenii* on YPD alone, were selected. Only hits mapped to non-overlapping ORFs with a homolog in other yeasts were analyzed further. Raw transcriptomic data is deposited at ENA under Primary accession # PRJEB23926, with a summary in S1 Data.

### GO term analysis

Subsets of genes and proteins from the proteomic and transcriptomic analyses were subjected to Gene Ontology (GO) term category analysis using FungiFun2 [54]. Only genes and proteins with a homologous *S*. *cerevisiae* or *C*. *albicans* gene were used. Proteins with a 10-fold abundance during starvation (SD) or predation (SD + *S*.*c*.) conditions, compared to standard conditions (YPD) were selected. Proteins were further divided as enriched during any of three conditions; “Starvation” (>10-fold higher abundance during starvation compared to predation conditions), “Predation” (>10-fold higher abundance during predation compared to starvation conditions) or “Predation + Starvation” (the rest). For the subsets of transcribed genes, genes with a total experimental range >100 and with a 10-fold upregulation during any of the seven experimental conditions, compared to *S*. *schoenii* on YPD alone, were selected. Upregulated genes were curated as primarily upregulated during three conditions; “Nutrient limitation” (>10-fold upregulated when on CSM alone, compared to when on YPD alone), “Methionine deprivation” (>2-fold upregulated when on CSM-Met alone, compared to when on CSM alone) and “Predation” (>2-fold upregulated when co-cultured with *S*. *cerevisiae* on any media, compared to when cultured alone on the same media). For the FungiFun2 analysis, the corresponding *C*. *albicans* homolog gene name for each gene was entered, using a background list of all *S*. *schoenii* genes with their *C*. *albicans* homolog. Significance of over-representation (enrichment) of direct GO terms was calculated using a Hypergeometric distribution test and adjusted with a Benjamini-Hochberg procedure (FDR correction).

## Results

### Contact-dependent mycoparasitism enables *S*. *schoenii* to eliminate *S*. *cerevisiae*

*S*. *schoenii* is one of the most efficient predator yeasts in the *Saccharomycopsis* clade and we first validated that *S*. *cerevisiae* was susceptible to predation by *S*. *schoeni* [25]. Just like *S*. *fibuligera*, *S*. *schoenii* is hygromycin sensitive and we therefore chose a hygromycin (hyg) resistant *S*. *cerevisiae* strain (*HSP104*::hyg) [44] as prey. We cultured *S*. *schoenii* and *S*. *cerevisiae HSP104*::hyg separately or in co-culture at equal ratios on starvation media (SD) for three days. Both *S*. *schoenii* and *S*. *cerevisiae* remained viable and able to form new colonies on standard media (YPD), whereas only *S*. *cerevisiae HSP104*::hyg could form colonies on hygromycin media (YPD + hyg) (S1 Fig). After being co-cultured with *S*. *schoenii*, a complete absence of *S*. *cerevisiae* colonies on YPD + hyg indicated that *S*. *cerevisiae* had been eliminated by *S*. *schoenii*.

**Fig 1:**
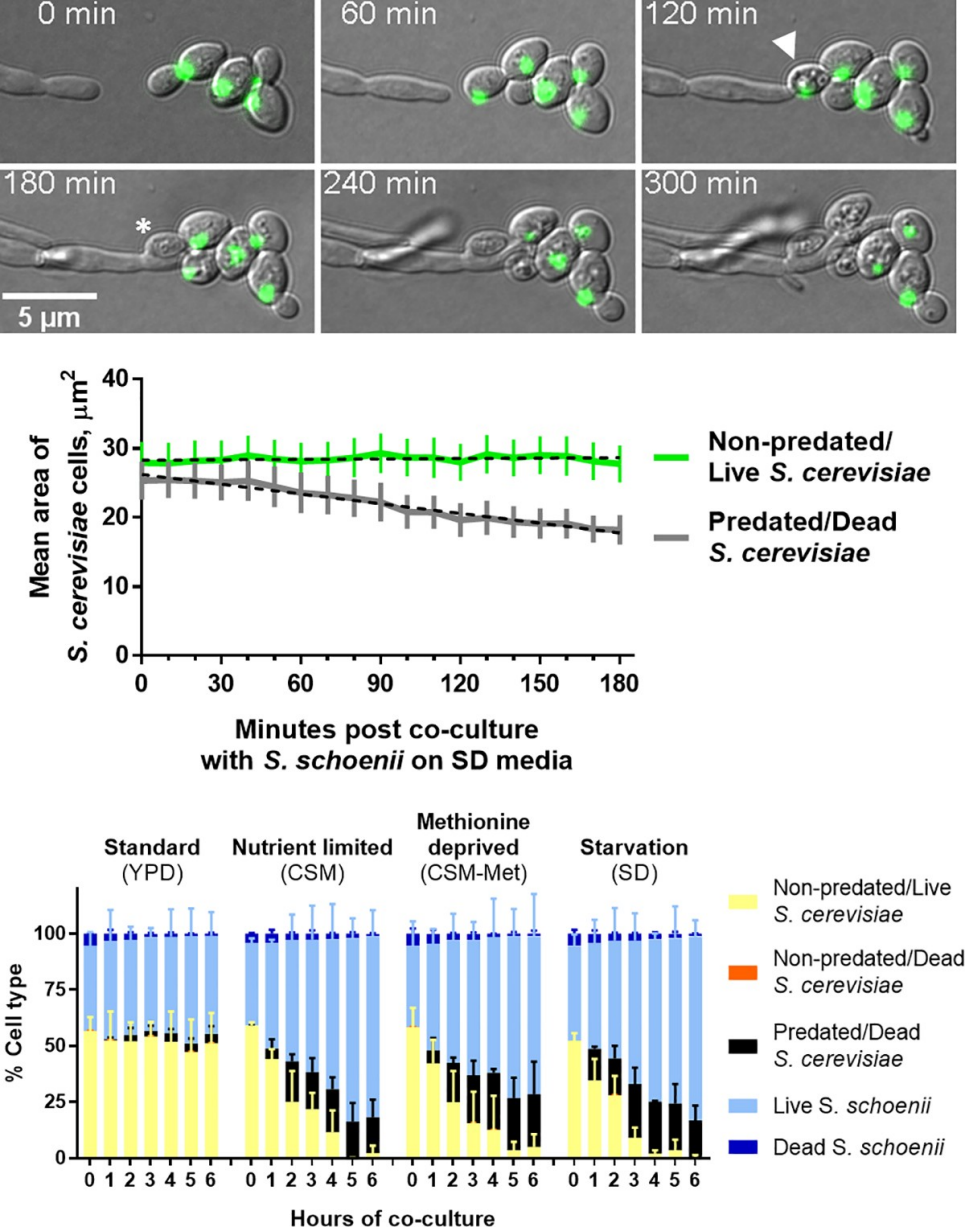
*S*. *schoenii* predates on and eliminates *S*. *cerevisiae*. A) *S*. *cerevisiae* (H4-GFP) cells collapse after contact-mediated mycoparasitism. Upon physical contact, *S*. *cerevisiae* cells vacuolarize (arrowhead), collapse in size and lose their H4-GFP signal (asterisk). B) The area of non-predated and predated *S*. *cerevisiae*. While non-predated cells stayed the same size, predated cells shrank significantly. Error bars = 95% CI. Dotted lines; linear regression of slopes, p-value of slopes <0.0001. C) Quantification and timing of predation during co-culture of *S*. *schoenii* and *S*. *cerevisiae* under different nutritional conditions. Cells were scored hourly on morphology-based viability. Results from two independent replicates, plotted as means with SD error bars.

In order to detect when and how *S*. *schoenii* eliminates *S*. *cerevisiae*, we co-cultured *S*. *schoenii* with a histone 4-GFP labelled *S*. *cerevisiae* strain (*H4*-GFP) on SD media and monitored cellular interactions with live cell microscopy (Fig 1A and S1 Movie). Upon physical contact with prey cells, predation by *S*. *schoenii* triggered the *S*. *cerevisiae* cells to first vacuolarize, then to shrink and lose their *H4*-GFP label (Fig 1A). Non-predated cells devoid of physical contact with *S*. *schoenii*, did not shrink nor lose their *H4*-GFP label and we observed a significant change in size between predated and non-predated cells (Fig 1B). In addition, we noticed that predated cells were unable to bud, whereas non-predated cells would bud continuously which supports previous observations that *S*. *schoenii* efficiently kills the model prey cell *S*. *cerevisiae* through contact-dependent mycoparasitism.

### Draft genome of *S*. *schoenii* reveals expansion of aspartic proteases

**Fig 2:**
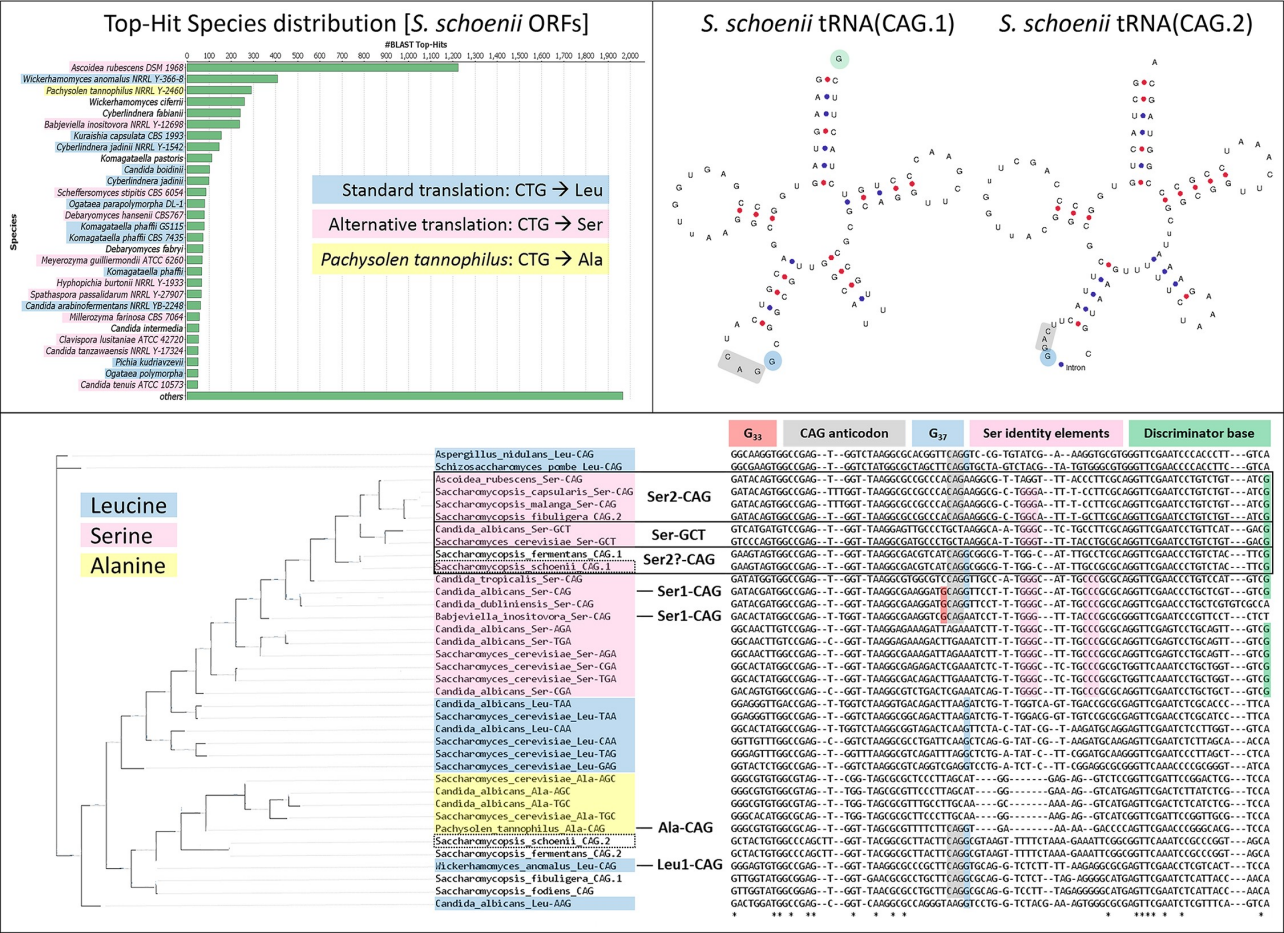
*In silico* analyses suggest *S*. *schoenii* is a CTG clade member. A) A cloudblast of translated *S*. *schoenii* ORFs against proteins suggested by nr database. *S*. *schoenii* was closely related to several CTG clade members. B) Two CAG-tRNAs were found in the draft *S*. *schoenii* genome. C) Alignment of the two *S*. *schoenii* CAG-tRNAs with CAG-tRNAs from *Saccharomycopsis fermentans* [28], *C*. *albicans*, *C*. *dubliniensis*, *C*. *tropicalis* as well as other Leu-tRNAs and Ser-tRNAs from *S*. *cerevisiae* and *C*. *albicans*. The *S*. *schoenii* CAG.2-tRNA aligns the closest with a *S*. *cerevisiae* Leu-GAG-tRNA, whereas *S*. *schoenii* CAG.1-tRNA aligns closer to the Ser-CAG-tRNAs of other CTG clade yeasts. CAG-tRNA features are color-coded [55–57].

In order to provide a genome with both good coverage over long repetitive stretches and with high fidelity, we sequenced the genome and assembled scaffolds with long PacBio reads, and subsequently polished the scaffolds with short 250-bp paired-end library and 8-kb mate-pair Illumina Miseq reads (Table 1A and S1 Material). We identified 7,999 open reading frames in the draft genome of *S*. *schoenii* (S2 Material). The non-redundant (nr) protein database contains protein sequences from all species deposited in GenBank, and we ran all ORFs through a cloud-based blastx strategy against the nr protein database (Table 1B). This provided us with a match for each ORF to the closest species, of those deposited in the nr database to date, permitting us to validate the close relationships between *Saccharomycopsis*, *A*. *rubescens* and *W*. *anomalus* (Fig 2A). Several, but not all, of the most similar yeast species were other CTG clade members. The number of *S*. *schoenii* ORFs with functional homologs to *S*. *cerevisiae* or *C*. *albicans* are listed in Table 1B. After we manually removed overlapping ORFs, the total number of genes with homologs came to 4,660. More ORFs were homologous to genes from *C*. *albicans* than to genes from *S*. *cerevisiae*, suggesting a closer relationship to *C*. *albicans* than *S*. *cerevisiae*. A number of non-overlapping ORFs were at least 100 amino acids long but lacked homologs from any of the blastx strategies.

**Table 1:** *schoenii de novo* draft genome assembly and annotation. ***S*.** A) *S*. *schoenii de novo* draft genome assembly. B) *S*. *schoenii* draft genome annotation. For subsequent functional analyses, the 4,660 genes with homologs were used. a) Identified with tRNA Scan-SE. b) ORF = min 300bp/100aa, ATG start site. c) using the Alternative Yeast Nuclear Code, translation table 12. d) Manual curation of non-overlapping genes. e) Blastx hit score >55. f) deposited by 06.06.17. g) non-overlapping ORFs, any blastx hit bit score >55. h) non-overlapping ORFs, blastx hit bit score <55. C) Gene loss in the sulfate assimilation pathway. D) List of gene expansion. Names of homologous *S*. *cerevisiae* or *C*. *albicans* genes.

**A) *S*. *schoenii* draft genome assembly**				**C) Lost sulfate uptake and reduction genes**				
Genome size (bp)			14 314 649	*MET1*	*MET3*	*MET5*	*MET8*	*MET10*
Contigs			29	*MET14*	*MET16*	*SOA1*	*SUL1*	*SUL2*
N50			1 481 735					
Total GC content			33.84%					
				**D) Selection of expanded genes**				**Copies**
**B) *S*. *schoenii* draft genome annotation**				*C4_03230C*	Transposable element gene			23
tRNA genes^a^			140	*CTS1*	Endochitinase			19
Total ORFs found^b^			7999	*TNA1*	Putative nicotinic acid transporter			19
	Translated^c^ non-overlapping^d^ ORFs with homology^e^ to			*FLO1*	Lectin-like protein involved in flocculation; cell wall protein that binds mannose chains on the surface of other cells			17
		*S*. *cerevisiae* proteins	4032	*YPS3*	Aspartic protease; member of the yapsin family of proteases involved in cell wall growth and maintenance			11
		*C*. *albicans* proteins	4281	*FLO9*	Lectin-like protein with similarity to Flo1p			7
		*S*. *cerevisiae* or *C*. *albicans* proteins	4427	*YBL100W-B*	Transposable element gene			6
		Proteins in the non-redundant protein database (nr) ^f^	4542	*CRH1*	Chitin transglycosylase			5
Genes with any homolog^g^			**4660**	*NRG1*	Transcription factor/repressor			5
	Transcribed genes		4646	*PDR12*	Plasma membrane ATP-binding cassette (ABC) transporter			5
Putative genes without any homolog^h^			263	*SEO1*	Putative sulfate compound permease			5
	Transcribed putative genes		261	*SIM1*	Adhesin-like protein; involved in cell wall maintenance			5

Just like we were unable to detect any genes in the sulfate assimilation pathway in the genomes of *S*. *fodiens* [27] and *S*. *fermentans* [28], the genes for sulfate uptake and reduction were all absent in the draft genome of *S*. *schoenii* (Table 1C). There were several highly expanded gene families in the *S*. *schoenii* draft genome, including aspartic proteases (*YPS3*), transposable elements, permeases and other cell wall related genes (Table 1D).

### *S*. *schoenii* harbors two tRNA(CAG) genes and translates CTG to serine

We were able to identify two tRNA(CAG) genes in the draft genome of *S*. *schoenii*, by running the draft genome sequence through tRNAscan-SE 2.0 [51] (Fig 2B). We were also able to identify two tRNA(CAG) genes in the genome of *S*. *fermentans*, but only one tRNA-CAG gene in the *S*. *fodiens* genome [27, 28]. We aligned these *Saccharomycopsis* tRNA(CAG) genes with serine and leucine tRNA genes from *S*. *cerevisiae* and *C*. *albicans*, as well as with tRNA(CAG) genes from other CTG clade yeasts, including yeasts from the recently proposed CTG subclades (Fig 2C) [55, 56]. The tRNA(CAG.1) genes of *S*. *schoenii* and *S*. *fermentans* were identical and aligned close to other Serine-tRNAs, whereas the also identical tRNA(CAG.2) genes aligned somewhat closer to leucine-tRNAs. The *S*. *schoenii* and *S*. *fermentans* tRNA(CAG.1) bore several hallmarks of the well described tRNA^Ser^(CAG), from *C*. *albicans*, including a m^1^G_37_ Leu-identity element and a (G_73_) discriminator base (Fig 2B) [57, 58]. Like *C*. *tropicalis*, neither *S*. *schoenii* nor *S*. *fermentans* tRNA-CAG.1 featured the leucylation-lowering G_33_. The tRNA(CAG.2) from *S*. *schoenii* and *S*. *fermentans* bore no serine-identity elements and were also less similar to other leucine-tRNA.

To find out how *S*. *schoenii* translated the CTG codon, we analyzed the *S*. *schoenii* proteome. Of the identified proteins that harbored a CTG codon(S1A Table), 450 had peptidic evidence of >1 CTG codon being translated to serine, whereas 13 were mapped to leucine (S1B Table). In *C*. *albicans*, around 3% of CTG codons are “mistranslated” to leucine, which incidence might be correlated with virulence [7]. To test whether or not *S*. *schoenii* varied its translation of the CTG codon during starvation and/or predation conditions, we cultured *S*. *schoenii* either alone under starvation (SD) or predation (SD + *S*. *cerevisiae*) conditions for three hours. During starvation, 14 proteins had CTG positions translated to leucine, whereas during predation, only 5 proteins had CTG positions translated to leucine (S1B Table). Three proteins (CET1, ZIM17 and ARO8) had peptides supporting translation of CTG to both serine and leucine under any one condition. Only one protein, *CAB3*, was translated only to serine during one condition (YPD) and leucine under another (SD).

### *S*. *schoenii* proteins involved in Starvation and Predation

**Fig 3:**
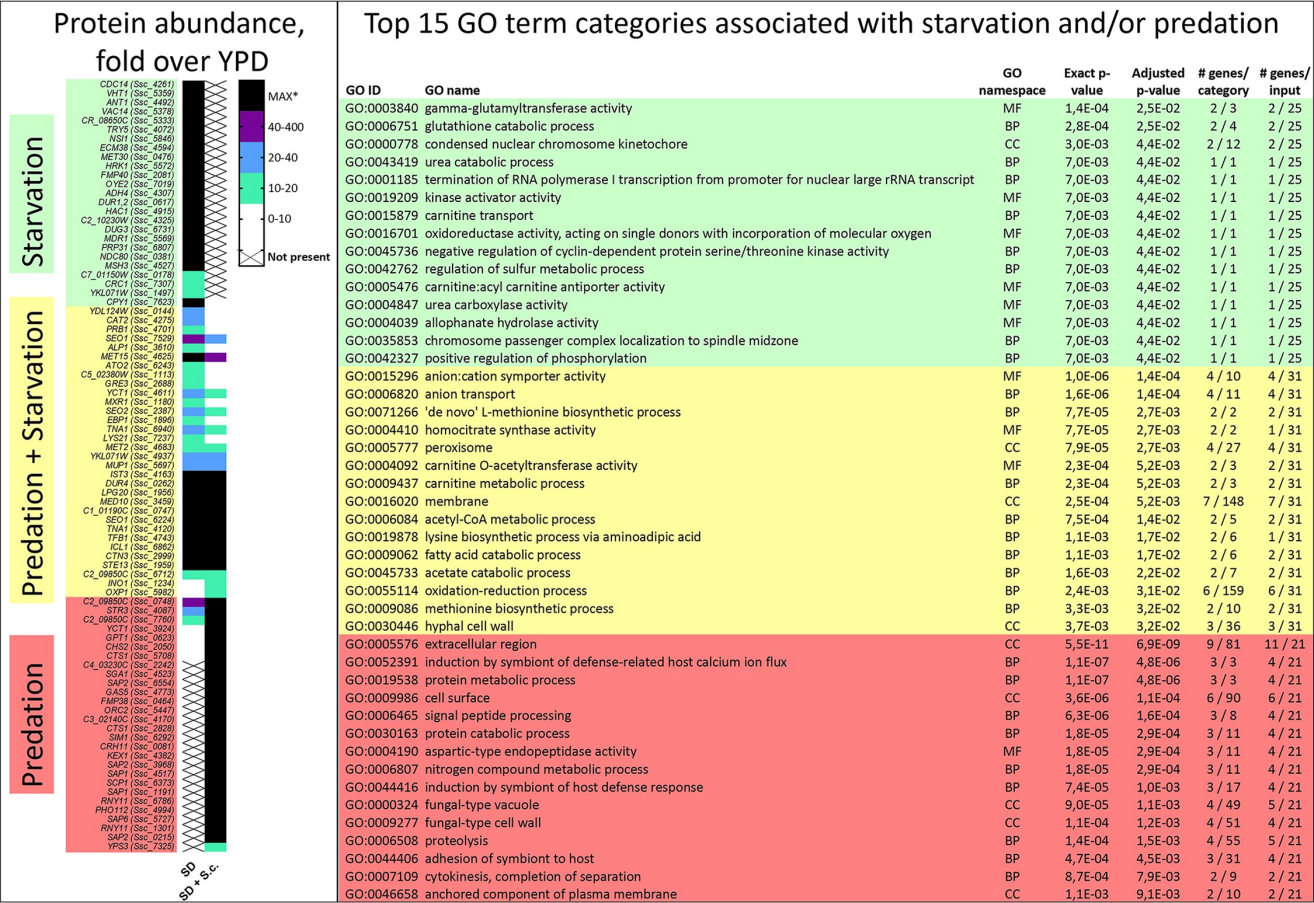
Proteins and GO term categories increased during starvation and/or predation conditions. A) Presence and relative abundance of *S*. *schoenii* proteins during starvation and predation conditions. Translated genes with homologs to either *S*. *cerevisiae* or *C*. *albicans*, with a protein abundance at least 10-fold higher during starvation conditions (SD) or predation conditions (SD + S.c.) were selected and listed with its corresponding gene ID. The proteins were curated into three subsets; proteins highly abundant under starvation (green), predation (red) or in both conditions (yellow). B) The top 15 GO term categories of *S*. *schoenii* proteins involved in starvation (green), predation (red) or abundant in both conditions, as output from FungiFun2 [54]. Proteins are analyzed in FungiFun2 by their gene name. #Genes/category defines how many genes can theoretically be found in each GO ID, whereas #genes/input defines how many of the submitted genes per total submitted genes belong to each GO ID.

Our next aim was to identify, quantify and categorize *S*. *schoenii* proteins present during starvation (SD) and/or predation (SD + *S*. *cerevisiae*), relative to standard (YPD) conditions. Protein and peptide concentrations were measured and analyzed and a protein abundance was expressed as the ratio of protein concentration compared to standard (YPD) conditions. We manually curated all proteins with at least 10-fold increase into subsets as relevant to either Starvation, Predation or both conditions (Fig 3A). To identify functionally meaningful enrichment patterns, we subjected the proteins in each subset to a GO term category analysis, using the online resource FungiFun2, against *C*. *albicans* proteins [54]. During Starvation, proteins involved in catabolic processes (Cis2, Dug3 and Dur1,2) as well as a protein regulating sulfur metabolic processes (Met30) were enriched. During Predation, several cell wall related proteins (Cht3, Crh11, Rny11, Sap1, Sap2, Sap6, Sim1, Pga4 and Pho112) were enriched (Fig 3B). During both starvation and predation, proteins in the biosynthesis of methionine (Met2, Met15 and Str3) were enriched, as well as transporters (Dal7, Fen2, Seo1 and Tna1), carnitine (Cnt3 and Cat2) and fatty acid catabolic (Icl1 and Cat2) related proteins.

### Nutrient limitation is the main trigger for predation

To quantify predatory activity, as a consequence of time and nutritional conditions, with specific regards to the absence or presence of methionine, we set up a microscopy-based predation assay. *S*. *schoenii* was able to attack and kill *S*. *cerevisiae* when co-cultured on all different nutritional conditions, including on the nutrient rich Standard media (Fig 1C). On Starvation media, *S*. *schoenii* nearly eliminated *S*. *cerevisiae* after 6 hours of co-culture. Non-predated/dead prey cells made up < 0.01% of all prey cells at any time or condition, reflecting “spontaneous” death of prey cells. “Spontaneous” death of *S*. *cerevisiae* similarly made up less than < 0.01% observed cells when cultured alone on any media, excluding any direct effect of the culture media on their viability.

To tease out if the absence of methionine would have any effect on the predatory propensity of *S*. *schoenii*, we co-cultured *S*. *schoenii* and *S*. *cerevisiae* on CSM and CSM-Met. CSM and CSM-Met are both nutrient limited media made up primarily of sugars and essential amino acids, which excludes cysteine, and differ only in the presence or absence of methionine. We were unable to detect any difference in the propensity or speed at which *S*. *schoenii* predated on *S*. *cerevisiae* in regard to the specific absence or presence of methionine, using this setup (Fig 1C). Instead, the greatest difference in predation activity was seen between YPD and CSM. Proliferation of *S*. *cerevisiae* when cultured alone, was not significantly affected between YPD and CSM during the 6 hours assayed, suggesting that the increased predatory activity was unlikely to primarily be a consequence of a less fit prey cell, but instead a response from *S*. *schoenii* to nutrient limited conditions.

### *S*. *schoenii* responds drastically to methionine deprivation

**Fig 4:**
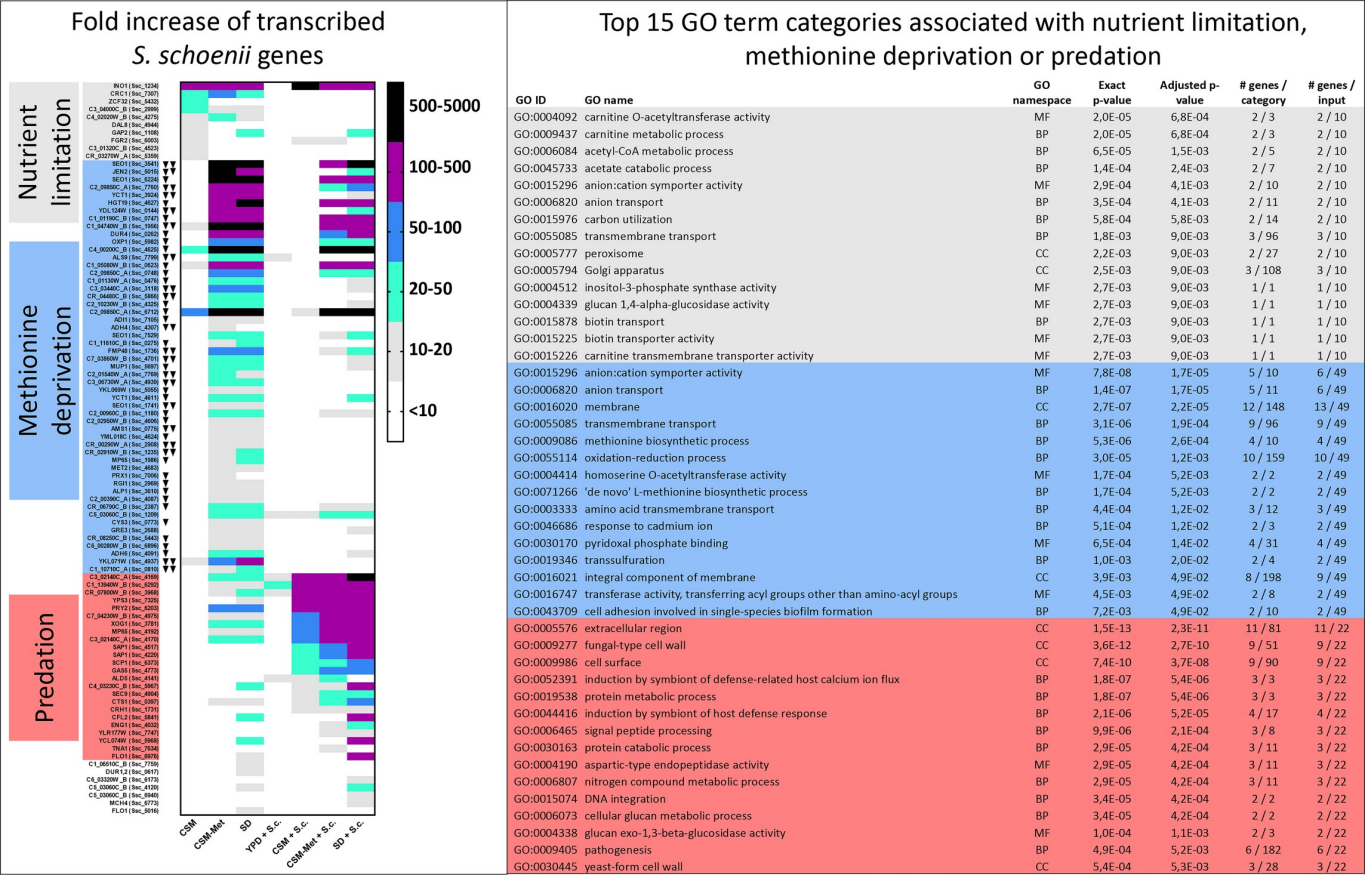
*S*. *schoenii* gene and GO term categories upregulated during gradual nutrient availability and/or predation conditions. A) Fold upregulation of *S*. *schoenii* genes during gradual nutrient availability, compared to *S*. *schoenii* on standard media (YPD), without (right) or with (left) *S*. *cerevisiae* present as prey. Upregulated genes were manually curated into three categories; mainly upregulated during conditions of Nutrient limitation (CSM, grey), of Methionine deprivation (CSM-Met, blue) or Predation (CSM + S.c., CSM-Met + S.c. or SD + S.c.). Down pointing arrows symbolize genes with two (one arrow) or five (two arrows) fold lower fold increase values when prey was present, compared to when no prey was present, on CSM-Met. B) Top 15 GO term categories of genes upregulated during Nutrient limitation, Methionine deprivation of Predation conditions. Genes are analyzed in FungiFun2 by their gene name. #Genes/category defines how many genes can theoretically be found in each GO ID, whereas #genes/input defines how many of the submitted genes per total submitted genes belong to each GO ID.

To identify which *S*. *schoenii* genes are involved in responses to nutrient limitation, methionine deprivation and starvation and to subsequently isolate the genes specifically involved in predation responses, we performed genome-wide transcriptomic analyses. We selected all genes with a 10-fold upregulation during any of the eight conditions, compared to *S*. *schoenii* on YPD alone and manually curated most of the upregulated genes into three subsets; genes upregulated mainly under conditions of “Nutrient limitation”, “Methionine deprivation” or “Predation” (Fig 4A).

To extract functionally meaningful enrichment patterns in the subsets of highly upregulated genes, we used the online resource FungiFun2, with a *C*. *albicans* gene nomenclature [54] (Fig 4B). During Nutrient limitation conditions, GO categories such as carnitine and acetyl-CoA metabolism (*CTN3*, *CAT2* and *CRC1*), anion and cation transport (*DAL8*, *VHT1* and *FGR2*) and the Golgi apparatus (*SGA1*, *GAP2*, *VHT1*) were highly enriched, suggesting a focus on peroxisome energy generation and membrane transport facilitation [59, 60]. During Methionine deprivation, membrane transport (*DAL7*, *YCT1*, *TNA1*, *FEN2*, *SEO1*, *GPT1*, *C1_10710C*, *MUP1*, *HGT19*, *TPO3*, *DUR4* and *JEN2*) GO terms were highly enriched, together with methionine biosynthesis (*CYS1*, *MET17*, *MET2* and *CYS3*) and oxidation-reduction processes (*C1_01190C*, *ADI1*, *MXR1*, *C2_01540W*, *ADH4*, *C2_09850C*, *GRE3*, *ADH6*, *PRX1* and *C7*_*03350C*) suggesting, in concert, extensive scavenging and salvaging efforts of methionine and other sulfur compounds (Fig 4B).

### *S*. *schoenii* probably acquires methionine from prey cells

**Fig 5:**
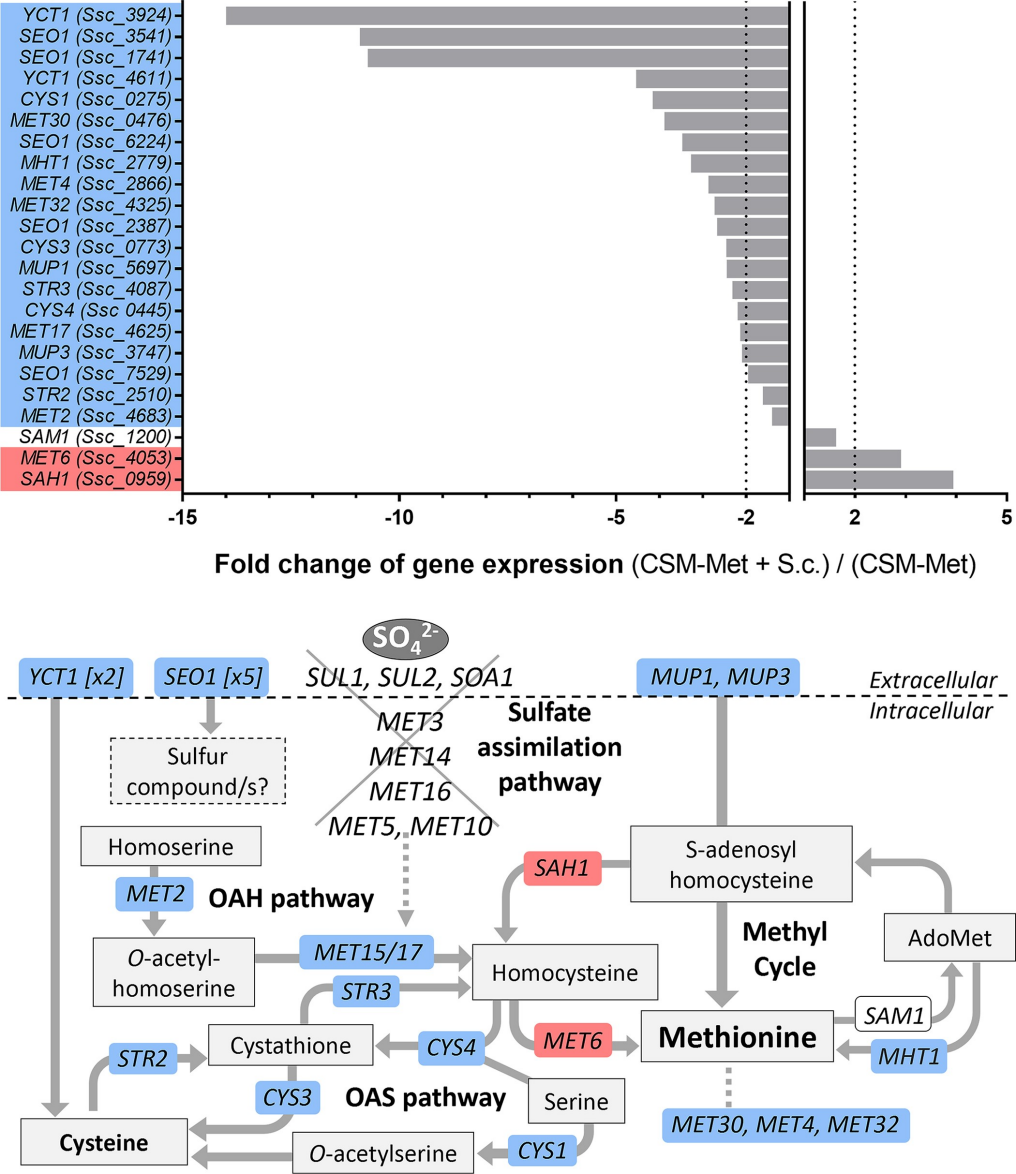
Methionine biosynthesis pathway and sulfur compound uptake genes in *S*. *schoenii*. Genes highlighted in blue were upregulated at least two-fold when methionine was missing compared to present (CSM/CSM-Met). Genes highlighted in red were upregulated at least two-fold when prey was present compared to absent, during methionine deprivation (CSM-Met+S.c./CSM-Met). A) Fold change of *S*. *schoenii* gene expression during methionine deprived conditions, when prey was present compared to absent (CSM-Met + S.c./CSM-Met). Dotted lines indicate two-fold changes up or down. B) *S*. *schoenii* lacks all genes in the sulfate assimilation pathway, but have two methionine permeases (*MUP1*, *MUP3*), two copies of the cysteine transporter *YCT1* and several copies of *SEO1*, a putative sulfur compound permease.

Interestingly, we noticed that the majority of *S*. *schoenii* genes that were upregulated in the methionine deprivation subset were downregulated at least twofold when *S*. *cerevisiae* was available as prey (Figs 4A and 5A). When we looked at the genes involved in methionine biosynthesis and uptake, all genes leading up to the methyl cycle and methionine, except *SAH1*, *MET6* and *SAM1*, were at least twofold upregulated when methionine was absent, suggesting increased need to scavenge and salvage of methionine and other sulfur compounds (Fig 5B). *SAH1* was the sole downregulated gene when methionine was absent. *MET6* and *SAH1* stood out as being upregulated at least twofold when the prey was present, suggesting that precursors needed to operate for the methyl cycle might have been acquired during predation.

### Aspartic proteases and glucanases are upregulated in *S. schoenii* during predation

During Predation, GO term categories relating to the cell wall surface (*XOG1*, *ENG1*, *SIM1*, *PRY2*, *MP65*, *CRH11*, *GAS1*, *SAP6*, *SAP1*, *SAP2*, *CHT3* and *ALS9*), were significantly enriched (Fig 4B). The most outstanding gene family was the secreted aspartic proteases (*SAP*) genes, homologous to yapsin (*YPS*) genes in *S*. *cerevisiae*. The aspartic protease genes were associated with several GO term categories, such as protein metabolic and catabolic process, signal peptide processing, aspartic-type endopeptidase activity and pathogenesis. Indeed, several *S*. *schoenii* aspartic protease genes were exclusively overexpressed when *S*. *cerevisiae* was present as prey, and correlated with predation efficacy (S2A Fig). Several aspartic protease genes were indeed found to be both expressed and translated to proteins (Figs 3 and 4). Overexpression of the glucanases *MP65*, *XOG1* and *ENG1*, the glycosidases *GAS1* and *CRH11* the chitinase *CHT3* also correlated with predation efficacy, but these were also upregulated, albeit to a lesser degree, when prey was absent (S2B Fig). Two transposable element genes, associated with DNA integration, *C4_03230C* and *YCL074W*, were upregulated during predation.

## Discussion

Yeasts in the *Saccharomycopsis* clade are potent necrotrophic mycoparasites of other yeasts, and *S*. *schoenii* has a specific potential as an antifungal biocontrol agent against human yeast pathogens, including multidrug-resistant *C*. *auris*. However, the molecular mechanisms involved in the predatory behavior have not been described for any *Saccharomycopsis* species. Identifying the genetic basis of their mode of action will facilitate both our basic understanding of their unique biology as well as enable prospecting of novel antifungal enzymes.

In this study, we integrated quantitative live cell microscopy assays with genomic, transcriptomic and proteomic approaches to identify genes and proteins that are overexpressed by *S*. *schoenii* during its predation of the model prey cell *S*. *cerevisiae*. We found high copy numbers of aspartic proteases in the *S*. *schoenii* genome, consistent with conclusions that mycoparasitic fungi typically harbor major gene expansion related to their parasitism [12]. We found that substantial overexpression of four aspartic protease genes correlated with predatory activity in *S*. *schoenii*, analogous to overexpression of SAP genes during *C*. *albicans* pathogenesis [61]. Surprisingly, despite the rare absence of genes in the sulfate assimilation pathway, general nutrient limitation, and not a specific presence or absence of methionine, appeared to be the main trigger of predatory activity in *S*. *schoenii*. However, the specific removal of methionine did not go unnoticed, as it triggered major transcriptional responses in *S*. *schoenii*, including upregulation and translation of *MET30*. Met30p might be part of a ubiquitin ligase that sense methionine and S-Adenosyl methionine (SAM) availability to the cell cycle control and transcriptional responses [62–64]. In *S*. *schoenii* the dual role of Met30p might therefore be to both increase protein degradation by the proteasome and to stop cell cycle progression until cellular levels of methionine are sufficiently high again. Interestingly, methionine-specific responses in *S*. *schoenii* were alleviated when prey cells were present, suggesting methionine might be acquired in some form from prey cells. In comparison, the basidiomycete *Puccinia striiformis*, also deficient in sulfate uptake, specifically upregulates S-methylmethionine permease in its haustoria during plant infection^8^.

Our finding that *S*. *schoenii* has lost all genes required for sulfate assimilation fits with the rare inability of *Saccharomycopsis* yeasts to assimilate sulfate [25]. While no other yeasts are unable to take up sulfate, three of the top ten most important parasitic filamentous fungi, *Puccinia spp*., *B*. *graminis* and *Melampsora lini* share this sulfate assimilation deficiency [65]. In addition, even parasites with functional sulfate assimilation, such as *Trichoderma* species, increase sulfur metabolism during their mycoparasitic activity [66]. This points to a convergent evolution of the ability of some yeasts and fungi to obtain sulfur from other sources than sulfate, probably associated with their parasitic abilities. Since methionine is the most energetically costly amino acid to biosynthesize [23] and several metabolites in the sulfate reduction pathway are toxic[24], it can be speculated that organic sulfur compounds are a top bounty for parasites. In this study, *S*. *schoenii* was able to nearly eliminate *S*. *cerevisiae* after just six hours of co-culture under nutrient limited conditions, demonstrating its potency as a prospective biocontrol agent. However, we had not anticipated that *S*. *schoenii* would predate on *S*. *cerevisiae* on nutrient rich media, pointing at *S*. *schoenii* being a facultative parasite not only for survival during stressful conditions, but also to actively eliminate competitors.

*Saccharomycopsis* yeasts were recently reassigned as a subclade in the CTG clade of yeasts and we confirmed this with our genomic and proteomic analysis of *S*. *schoenii* [20]. Just like Krassowski *et al*. [20] found in other *Saccharomycopsis* species, we identified two tRNA(CAG) genes in the *S*. *schoenii* genome. We conclude that one must be a tRNA^Ser^(CAG), since our proteomic analysis supported translation of the CTG codon to serine. We cannot determine if the other tRNA(CAG) is functional, but even though we found that CTG codons were “mistranslated” to leucine 1% of the time, just like in *S*. *capsularis*, we are at this point unable to call any meaning or real significance to the “mistranslation” of these proteins.

We detected upregulation of two TEs during predation, but not nutrient limited conditions of *S*. *schoenii*. This could imply that these transposable elements might have roles in either protecting the genome of *S*. *schoenii* during predation, in altering gene expression, or have roles in silencing any defense mechanisms in the prey cells.

A limitation of this study is that we are only able to demonstrate correlation between gene expression and abundance with responses to nutritional stresses and predatory activity, not causation. For instance, with this setup we cannot prove if the overexpressed aspartic proteases and glucanases are destroying prey cell walls, or are used in remodeling the *S*. *schoenii* cell wall during predation. However, similar transcriptome based studies on pathogen associated genes have been validated in *C*. *albicans* [67]. With target genes potentially involved in predation at hand directed gene-function analyses will in the future provide deeper insight into the actual mechanisms employed during predation.

Our findings suggest that *S*. *schoenii* acquires methionine and other sulfur compounds from its prey cells. Further studies, enabled by for instance the knock-in of fluorescent tags, could determine whether the upregulated organic sulfur permeases *MUP1*, *SEO1* and *YCT1* are specifically distributed to the site of prey penetration. In addition, any role of cysteine as a trigger for predation should be considered. To determine if *S*. *schoenii* attacks all prey species with the same set of tools, or if its expanded set of aspartic proteases and glucanases act more like a Swiss army knife, adapted to specific prey cells, future studies using different prey species are needed.

In summary, our study sets the framework for further studies on the use of *Saccharomycopsis* yeast as potential biocontrol agents. We honed in on the timing and nutritional conditions under which *S*. *schoenii* kills the model prey species *S*. *cerevisiae*, identified and functionally characterized the genes and proteins involved during the predatory behavior of *S*. *schoenii* and provided a multi-omic foundation for further exploration of the ecology and evolution of *Saccharomycopsis* yeasts.

## Supporting information

S1 Fig *S. schoenii* can eliminate *S*. *cerevisiae*. Hygromycin sensitive *S*. *schoenii* cells and hygromycin resistant *S*. *cerevisiae* cells were cultured alone or co-cultured on SD media, and subsequently stamped onto YPD and YPD with hygromycin. After co-culture, no live *S*. *cerevisiae* is left, as indicated by no growth of *S*. *cerevisiae* on YPD + hygromycin (red box).
(TIF)

S2 Fig Correlation between percentage of predated *S*. *cerevisiae* cells (black line with circle) and overexpression of *S*. *schoenii* genes, after 3 hours of co-culture. A) Transcription values of yapsin/aspartic protease genes during co-culture of *S*. *schoenii* and *S*. *cerevisiae* in red, and during sole culture of *S*. *schoenii* in dotted black. B) Transcription values of glucanases, glycosidases and chitinase genes during co-culture of *S*. *schoenii* and *S*. *cerevisiae* in green, and during sole culture of *S*. *schoenii* in dotted black.
(TIF)

S1 Data *S*. *schoenii* omic data. *S*. *schoenii* genome and annotated genes plus transcriptomic and proteomic data from this study.
(XLSX)

S1 Movie *S*. *cerevisiae* (H4-GFP) cells collapse after *S*. *schoenii* exerts contact-mediated mycoparasitism. Movie version of Fig 1 (white insert).
(MP4)

S1 Table CTG translation in *S*. *schoenii*. Predicted and actual translation of CTG positions in *S*. *schoenii* genes.
(DOCX)

S1 Material *S. schoenii* draft genome sequence. (FA)

S2 Material *S*. *schoenii* draft genome annotation. (GFF)
