# Supplementary figures and images for: Multi-omics characterization of the necrotrophic mycoparasite Saccharomycopsis schoenii
Source: PLoS Pathog. 2019 May 9;15(5):e1007692. doi: 10.1371/journal.ppat.1007692 (PMC6508603; doi:10.1371/journal.ppat.1007692)

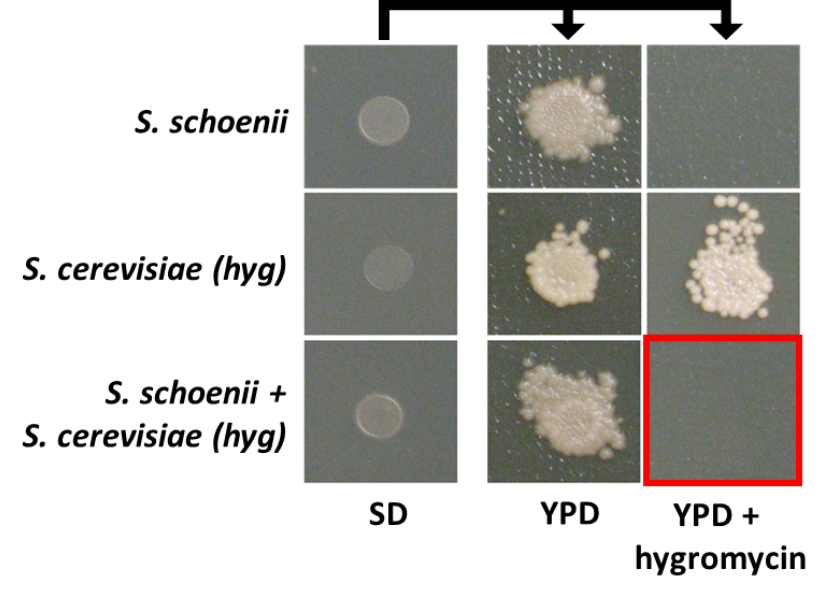

Supplement: S1 Fig — Hygromycin sensitive S. schoenii cells and hygromycin resistant S. cerevisiae cells were cultured alone or co-cultured on SD media, and subsequently stamped onto YPD and YPD with hygromycin. After co-culture, no live S. cerevisiae is left, as indicated by no growth of S. cerevisiae on YPD + hygromycin (red box). (TIF) [file ppat.1007692.s001.tif]

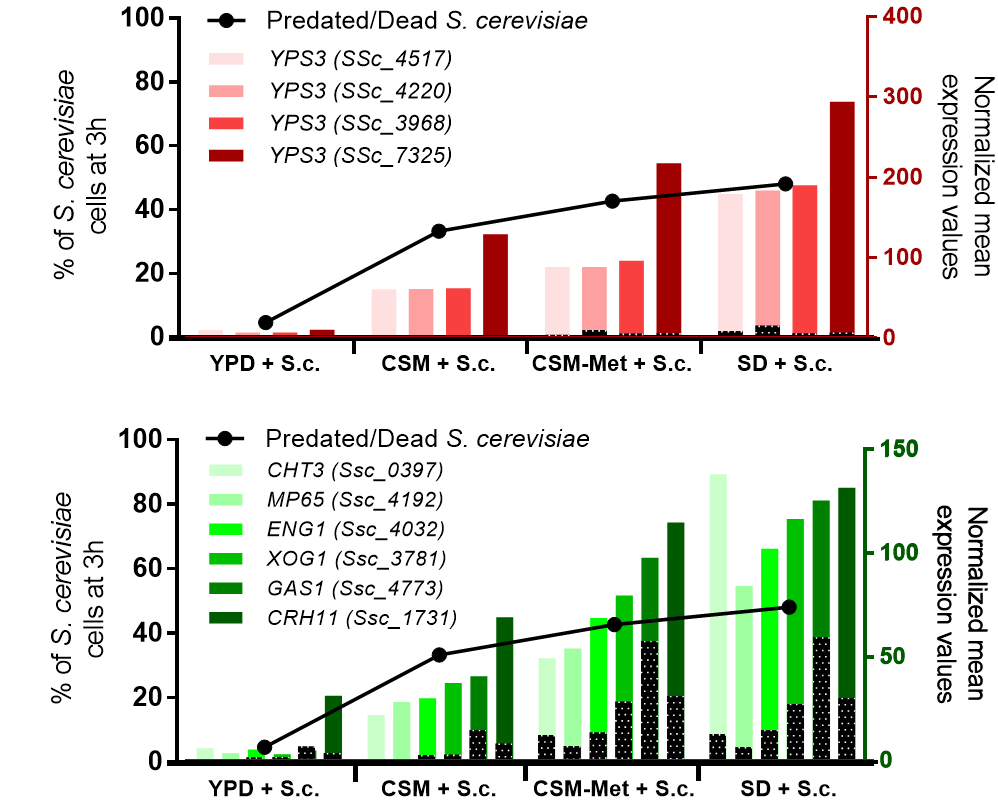

Supplement: S2 Fig — A) Transcription values of yapsin/aspartic protease genes during co-culture of S. schoenii and S. cerevisiae in red, and during sole culture of S. schoenii in dotted black. B) Transcription values of glucanases, glycosidases and chitinase genes during co-culture of S. schoenii and S. cerevisiae in green, and during sole culture of S. schoenii in dotted black. (TIF) [file ppat.1007692.s002.tif]
