# Supplementary material for: Multi-omics characterization of the necrotrophic mycoparasite Saccharomycopsis schoenii
Source: PLoS Pathog. 2019 May 9;15(5):e1007692. doi: 10.1371/journal.ppat.1007692 (PMC6508603; doi:10.1371/journal.ppat.1007692)
Supplement: S1 Table — Predicted and actual translation of CTG positions in S. schoenii genes. (DOCX) [file ppat.1007692.s005.docx]

| ***In silico* prediction of CTG codons in *S. schoenii* draft genome** | | | | |
| --- | --- | --- | --- | --- |
| Predicted genes (with any homolog) | | | | 4,660 |
|  | Predicted genes with CTG codons | | | 3,137 (67 %) |
|  | |  | Total number of CTG codons | 9,744 |
|  | |  | Genes with ≥6 CTG codons | 437 |
|  | |  | Genes with 3-5 CTG codons | 954 |
|  | |  | Genes with 1-2 CTG codons | 1,748 |
|  | | Genes with 0 CTG codons | | 1,523 (33%) |

| **Peptide evidence of CTG codon translation in *S. schoenii*** | | | | | |
| --- | --- | --- | --- | --- | --- |
|  | | | **Standard**  (YPD) | **Starvation**  (SD) | **Predation**  (SD + *S. cerevisiae*) |
| Proteins (with any homolog) identified by peptides | | | 2,609 | 3,009 | 2,876 |
|  | Identified proteins with ≥1 CTG codon | | 1,521 | 1,486 | 1,354 |
|  |  | ≥1 CTG → Leucine | **13 (0.8 %)** | **14 (0.9 %)** | **5 (0.4 %)** |
|  |  | ≥1 CTG → Serine | **450 (29.6 %)** | **430 (28.9 %)** | **399 (29.5 %)** |
|  |  | No CTG codon covered by peptide | 1,059 | 1,043 | 950 |
